# 低剂量螺旋CT肺癌筛查研究进展

**DOI:** 10.3779/j.issn.1009-3419.2013.10.10

**Published:** 2013-10-20

**Affiliations:** 1 300192 天津，天津市核医学重点实验室，中国医学科学院放射医学研究所，北京协和医学院 Peking Union Medical College & Institute of radiation medicine, Chinese Academy of Medical Science, Tianjin 300192, China; 2 300052 天津，天津市肺癌转移与肿瘤微环境重点实验室，天津肺癌研究所，天津医科大学总医院 Tianjin Key Laboratory of Lung cancer Metastasis and Tumor Microenvironment, Tianjin Lung cancer Institute, Tianjin Medical University General Hospital, Tianjin 300052, China

**Keywords:** 低剂量螺旋CT, 肺肿瘤, 筛查, Low-dose spiral CT, Lung neoplasms, Screening

## Abstract

肺癌已经成为全球范围内癌症死亡的首位死因，其5年生存率很低。肺癌的筛查和早期诊断是改善肺癌生存，降低肺癌死亡率的关键。低剂量螺旋CT是近20年肺癌筛查的热点，随机对照试验研究已经证实其可降低肺癌死亡率。但目前低剂量螺旋CT筛查仍存在一些问题。本文对近年来低剂量螺旋CT能否降低肺癌死亡率的争议、随机对照试验的结果及存在的问题进行综述，并对未来低剂量螺旋CT在肺癌筛查中的应用做一展望。

## Abstract (en)

Lung cancer has become the leading cause of cancer mortality globally, and 5-year survival rate is very poor. Screening and early detection are vital to improve survival and decrease mortality of lung cancer. In recent 20 years, low-dose spiral CT (LDCT) screening has become a research focus in this area. Randomized controlled trials have confirmed that LDCT can decrease lung cancer mortality. However, there are still some problems of LDCT. In this paper, we summarized the controversy that whether low-dose helical CT screening can reduce lung cancer mortality or not before its effectiveness was been confirmed, the results and problems in the randomized controlled trials and gave a prospect of low-dose helical CT screening's future application.

## 1 肺癌筛查基本现状

### 1.1 肺癌筛查的必要性

肺癌是全球范围内最常见的恶性肿瘤，其死亡是癌相关死亡的首位原因，2008年肺癌的发病和死亡分别占所有癌症发病和死亡的12.7%和18.2%^[1]^。在中国，无论是男性还是女性，城市或乡村，肺癌死亡率居癌症死亡的首位^[2]^。早期肺癌并没有明显的临床症状，而大部分患者在有临床症状时才去就诊，但病变确诊后，基本为中晚期肺癌，而其中仅有不到25%的患者适合手术治疗^[3]^，确诊的肺癌病例的5年生存率很低^[4]^。

吸烟是肺癌最重要的危险因素，中国男性75.04%的肺癌死亡可归因于吸烟^[5]^。控烟是肺癌一级预防的重要内容。但目前我国的控烟效果并不理想，短时间内烟草流行率不会明显下降。此外，长期吸烟者戒烟后肺癌发病危险仍高于从不吸烟者^[6]^。因此，作为肺癌的二级预防措施，肺癌的筛查和早期诊断对于改善肺癌生存，降低死亡率具有重要意义。

### 1.2 肺癌筛查高危人群的选择

肺癌筛查主要在高危人群中进行。目前各研究对高危人群的入选标准并不一致，主要是根据年龄和吸烟情况。在法国的Blanchon等^[7]^肺癌筛查研究中，研究对象为50岁-75岁的无症状，且当前吸烟（每天吸烟大于15支，持续20年）或者之前有吸烟史（戒烟不超过15年）的男性或者女性人群。而在纽约ELCAP项目中，研究对象为年龄60岁以上，吸烟史为10包/年的人群^[8]^。

PLCO肺癌研究通过*Logistic*回归模型，模拟年龄、性别、种族、教育水平、BMI指数、家族史、吸烟史等多个影响因素，模型中还考虑了性别种族间的交互作用。研究显示PLCO的人群选择模型较NLST的纳入标准的灵敏度和阳性预测值均获得改善。因此，肺癌高危人群模型可能有助于更准确的筛查高危人群，未来危险预测模型的建立可能需要考虑年龄和吸烟外更多的因素，如家族史等^[9, 10]^。英国2012年开展的肺癌筛查研究采用的模型为LLP（利物浦）风险模型^[11]^，模型不仅考虑了吸烟情况而且整合了年龄、性别、职业暴露等危险因素，研究对象的风险超过5%就会被纳入标准进行筛查。

## 2 LDCT螺旋肺癌筛查

### 2.1 肺癌筛查背景

肺癌筛查开始于20世纪50年代，Mayo肺癌筛查项目（Mayo lung proLP）^[12]^是早期被认为最有权威性的研究，研究对胸片X线检查（CXR）联合痰细胞学检查与非筛查组进行了比较，结果表明患者生存率有所提高，但并没有发现两组的肺癌死亡率存在统计学的差异^[13]^。胸片X线胸片检查，敏感性和特异性不高，容易漏诊或误诊；而痰细胞学检查，仅对早期累及大气管及支气管的鳞癌较为敏感^[14]^。美国前列腺、肺、结直肠和卵巢癌筛查试验（PLCO）结果显示：在随访13年后，X线胸片和常规治疗组的肺癌累积发病率分别为20.1/10, 000人年和19.2/10, 000人年，相对危险度为1.05（95%CI: 0.98-1.12），提示年度性胸片筛查不能降低肺癌死亡率^[15]^。另外肺癌早期标志物也是目前研究的热点，但目前绝大部分早期肺癌标志物缺乏前瞻性研究和随机对照试验的验证，尚不能应用于人群的筛查。

90年代Naildich等提出了用低剂量螺旋CT（low-dose spiral CT, LDCT）作为肺癌筛查的新方法。研究表明放射线剂量与管电流成线性关系，低剂量即在其他参数不变的情况下，降低管电流，从而降低放射剂量。由于肺组织本身的天然高度对比度和对X线的低吸收性，肺部的低剂量螺旋CT筛查是完全可能的^[16]^。

### 2.2 LDCT肺癌筛查效果

自20世纪90年代以来，利用低剂量螺旋CT进行肺癌的筛查研究广泛开展。早期的研究设计为无对照的队列研究，主要为评价低剂量螺旋CT对早期肺癌的检出能力和检出肺癌的生存情况。有研究表明，低剂量螺旋CT的筛查阳性率为X线胸片的3倍，检出肺癌能力为X线胸片的4倍，检出Ⅰ期肺癌能力为X线胸片的6倍^[17]^。低剂量螺旋CT筛查检出的肺癌病人生存也显著改善，国际早期肺癌合作研究组（I-ELCAP）低剂量螺旋CT筛查的结果显示，临床Ⅰ期肺癌的10年生存率可达88%，而经过手术治疗的Ⅰ期病例的10年生存率则达到92%^[18]^，作者因此认为低剂量螺旋CT可避免80%肺癌的死亡但由于该试验并非随机对照试验，学者对结果的可靠性提出了质疑。2007年Bach等^[19]^的研究发现单纯的LDCT肺癌筛检在肺癌诊断和肺癌切除上明显高于预期值（144 *vs* 44.5；109 *vs* 10.9），结果有统计学意义；但LDCT肺癌筛查在降低肺癌的恶化和减少肺癌死亡上并没有明显效果。

就循证医学角度而言，随机对照试验是评价肺癌筛查效果的证据最强的研究方法。DANTE等^[20, 21]^肺癌筛查研究中虽然筛查有利于肺癌的检出，但该试验并没有得到LDCT筛查能够降低肺癌死亡率的预期结果。同时期欧洲进行的几项肺癌随机对照筛查试验均是将筛查组与非筛查群进行比较来验证肺癌筛检是否能够降低肺癌的死亡率。ITALUNG研究，丹麦进行的DLSCT肺癌随机对照实验，德国肺癌筛查研究（LUSI）^[22-24]^，均未得出CT筛查能够减少肺癌死亡率的证据，这是由于样本量较小，其统计学效力不足以检出肺癌死亡率的降低。

随机对照实验中，研究样本量较大的是NELSON研究^[25]^和美国肺癌筛查实验（NLST）^[26]^。NELSON研究中将LDCT组与常规护理组比较，随访达到10年，到目前为止NELSON最终的死亡率分析结果还未公布。NLST试验随访均数为6.5年，涉及人数为53, 454人，LDCT筛查组肺癌死亡率为247人/10万人年，CXR筛查组为309人/10万人年，统计结果显示LDCT筛查组比CXR筛查能够降低20%的肺癌死亡率（RR=0.8; 95%CI: 0.73-0.93），总死亡率降低6.7%（RR=0.93; 95%CI: 0.86-0.99）总死亡率降低的主要原因是肺癌死亡减少导致的。研究中发现，要减少1例肺癌死亡需要筛查的人数为320人。这是首次发现的LDCT筛查能够降低肺癌率的证据。

### 2.3 LDCT筛检存在的问题

#### 2.3.1 假阳性

LDCT肺癌筛查能够有效地发现Ⅰ期肺癌及非小细胞肺癌，但是LDCT筛查在发现恶性结节的同时，也检出了大量良性和性质难以确定的结节，导致了很高的假阳性率。很多假阳性结节需要接受进一步的侵袭性检查，此外也增加了受试者的焦虑心理。*Meta*分析显示6个随机对照实验基线筛查中干预组的假阳性的危险为对照组的3.1倍^[27]^。

较高的假阳性率是多数的研究面临的共同的问题。另外大多数研究仅给出了第一轮的筛查结果，在NLST试验中三轮肺结节筛查阳性率分别为27.3%、27.9%和16.8%，总的阳性筛查率为24.2%（CXR组为6.95%）。但是阳性结果中有96.4%为假阳性^[26]^。

研究显示过高筛查阳性率越高，人群所体验的不舒适感越强烈，同时也越容易焦虑^[28]^。van den Bergh等^[29]^通过调查问卷的方式来研究不确定性的筛查结果是否会影响受试者短期健康生命质量（HRQoL）。研究发现在不确定的筛查结果得出的2个月后发现肺癌特异性痛苦感（Lung cancer-specific distress）明显高于前2个基线调查时期。而在长期效应研究中发现这种焦虑或影响并未持续下去^[30]^。目前并没有研究显示肺癌筛查阳性与否能影响研究对象的生存质量。研究者并没有发现知情同意的研究者与没做出知情同意者之间的HRQoL存在差别^[31]^。

#### 2.3.2 过度诊断

日本一项肺癌筛查研究中用检测和发生率的方法估计了LDCT肺癌筛检和CXR筛检的灵敏度和特异度。检测方法计算的LDCT组灵敏度为88.9%，特异度为92.6%，相对于CXR组为78.3%和97%；但是用发生率的方法得出LDCT组的灵敏度为79.5%，CXR为86.5%，这表明过度诊断在LDCT筛查中起到了一定的作用^[32]^。

Bach等^[19]^在评估LDCT筛查的结果时得出：3, 246例筛查者中，肺癌筛查确诊了144例肺癌患者，而模型预期仅有44.5例（*P* < 0.001）；109例患者进行肺结节切除，预期值为10.9（*P* < 0.001）；而筛查对进展期的肺癌以及肺癌死亡情况没有明显作用（*P*=0.9）。LDCT筛查的过度诊断增加了肺癌的检出率，同时也增加了肺结节的切除率。

估计过度诊断的作用最可靠的方法是长期的随机对照实验（RCT），对平行对照组的长期随访，可以将各组的发病率进行比较^[33, 34]^。Mayo肺癌研究中筛检组有206人被诊断，对照组有160人，筛检组比对照组筛检人数高出22%，作者认为高出的病例为无症状的肺结节患者。Marcus等^[35]^文章指出如果LDCT筛查有更高的灵敏度，那么LDCT筛查所导致的过度诊断率应该更高。然而在NLST中两组诊断例数仅有13%的差别，鉴于随访时间较短目前还不能得出过度诊断是否真的存在差异，同时PLCO中也发现筛查组与对照组仅有4.6%的差异，这远低于研究预期值^[33]^，目前的数据没有说明过度诊断的作用和危害。

#### 2.3.3 LDCT筛检的辐射风险

LDCT肺癌筛检另外一个有害因素是辐射暴露。LDCT的剂量比普通CT要低，但是受检个体要经历几轮的小剂量照射，这种小剂量照射的风险对研究对象的远期效应并没有精确的定量估计，并且辐射和吸烟的交互影响能够增加患癌风险^[36]^。2008年美国癌症研究所的研究^[37]^显示筛检所带来的死亡率的减少大于辐射带来的危害，但当LDCT应用于一般人群时，增加的癌症人数是不可忽略的。

LDCT肺癌筛检中，不同个体间所接受的剂量并不完全相同，因为筛查结果不确定的个体，就需要重复进行LDCT筛查或者更高剂量的CT筛查。另外不同研究中的仪器设定的不同，也使肺癌高危人群接受的剂量大不相同，因此不同个体的受照剂量的估计是目前面临应解决的一个问题。

### 2.4 肺结节的检查与处理

肺结节的诊断主要是根据其大小和水平，来进一步判断是否需要做下一步的肺癌诊断以及是否需要进行重复的LDCT筛查。随机对照实验中，基线肺结节的筛查率一般为2%-30%，队列研究中一般为5%到51%^[23]^。在NLST研究中新生结节≥4mm时定义为筛查阳性，而在NELSON中新生结节 > 500 mm^3^时被认为是筛查阳性，目前对结节的判断和处理各个研究不尽相同。不同筛查研究中结节的处理原则^[20]^见表 1。

**1:** 三组不同筛查试验中结节处理原则 Nodules management principles in three groups of different screening trials

Non-calcified solid nodule		Fleischner guidelines 2005	NLST	NELSON
Volume (mm^3^)	Diameter (mm)			
< 50	< 4	CT at 12 months; If unchanged, no further follow-up	None	LDCT at 12 months
50-500	4	CT at 6-12 months, then 18-24 months if unchanged	LDCT at 3, 6, 12 or 24 months, depending on lesion size and level of suspicion of malignancy	LDCT at 3 months
	5			
	6			
	7	CT at 3-6 months, then 9-12 months, and 24 months if unchanged		
	8			
	9			
	10			
> 500	> 10	CT at 3, 9 and 24 months If unchanged, PET, dynamic contrast-enhanced CT, and/or biopsy	LDCT at 3, 6, 12 or 24 months, depending on lesion size and level of suspicion of malignancy; and/or biopsy PET, dynamic contrast-enhanced CT	Referral to pulmonologist for work-up

另外，NELSON试验研究中首次加入了体积倍增时间（VDT）用来辅助判断肺结节的处理。VDT软件的使用减少了人为判断因素的偏倚，这是以后随机对照试验值得借鉴的地方。

### 2.5 LDCT筛检对吸烟的影响

经历LDCT肺癌筛查的患者，可能会继续吸烟或者选择性吸烟，这或许是为克服对筛检的结果的不安造成的。不过目前并没有足够证据证明LDCT筛检能够影响戒烟率。NELSON实验中对550名男性吸烟者并且检查阴性的研究对象进行了问卷调查与440名结果不确定的男性吸烟者的调查结果进行比较，二者在戒烟上没有明显差异（*P*=0.26）^[38]^。但也有一些非对比性的研究显示，研究对象接受LDCT肺癌筛检能够对这些吸烟人群的戒烟产生一定的好处。这些研究显示通过LDCT肺癌筛检，吸烟者接受LDCT筛查后戒烟情况均有所提高，戒烟率均在10%以上^[39]^，而CT筛检结果异常或者高度怀疑的研究对象戒烟率更高^[40]^。

### 2.6 LDCT筛检的成本效益

LDCT筛查相对于非筛查减少了大量的肺癌死亡。为挽救一条生命或者增加生命质量调整年并不确定，CT筛查肺癌的成本效益并不确定。到目前为止，只有高危人群即现在吸烟者或者有吸烟史的人群被选入研究中，但是在吸烟者的一生中仅有11%的女性和17%的男性吸烟者被诊断为肺癌。高危人群以及筛查时间间隔的分层筛选，有助于降低假阳性率，这也许会有助于分析肺癌风险增加肺癌的成本效益^[41]^。

Goulart等^[42]^根据国民健康访问调查数据及NLST的结果，对LDCT筛查的经济学分析，发现当美国符合LDCT筛查标准的高危人群参与率为50%-70%时，将增加13亿或者20亿的支出，参与率为75%时，每年将会减少8, 100例肺癌患者过早死亡，使用LDCT肺癌筛检来避免1例肺癌死亡的成本是240, 000美元成本效益率随模型变化范围很大，ELCAP的数据被纳入一个决策分析模型，来比较LDCT筛查与非筛查结果。单纯的基线LDCT筛查的成本效益率为避免一人死亡肺癌所需成本为2, 500美元，只有当过度诊断的可能性大于50%时，成本效益率才会超过50, 000美元/每人^[43]^。相比之下澳大利亚年龄在60岁-64岁的男性成本效益模型（肺癌发病率为552/10万人年），显示成本效益率为57, 325 AU/LY（life-year），生命质量调整年为105, 090 AU，女性为51, 001 AU和88, 583 AU^[44]^。美国就目前的数据估计LDCT肺癌筛检每年能够潜在地避免8, 000-12, 000例肺癌患者的死亡^[45]^，但过高的成本必然会让政府重新考虑它的实际应用价值，这个弊端必然也会限制LDCT的应用。

## 3 LDCT肺癌筛查指南

在NLST研究证实低剂量螺旋CT可降低肺癌死亡率之前，医学组织并不推荐肺癌的人群筛查。NLST研究结果发布以后，各医学组织也相应更新了肺癌筛查指南的内容。

美国癌症组织（American Cancer Society, ACS）^[46]^建议在进行首次肺癌筛查时，肺癌筛查者、临床医生以及治疗中心在评估肺癌筛查对象时应该考虑LDCT筛查的优势，不确定性以及危害；建议LDCT筛查的高危人群为55岁-74岁的现在吸烟或之前吸烟者（戒烟不超过15年）其中吸烟量为30包/每年。美国肺癌协会（American Lung Association, ALA）^[47]^除建议使用NLST筛查标准外，还提出戒烟并不能够避免肺癌风险，筛查不应被视为戒烟的替代品，筛查并不能检出所有肺癌，筛查的目的是预防肺癌；筛查过程中应告知筛查者LDCT筛查的利弊，风险以及成本，同时应该形成肺癌筛查公共卫生资料协助医生和筛查者间的交流。美国胸外科协会（American Association for Thoracic Surgery, AATS）^[48]^则呼吁，肺癌长期患者应每年进行LDCT筛查以检测第二原发性肺癌直到79岁，并建议如果人群5年的累积患肺癌风险大于5%时，就应该从50岁开始筛查。美国胸内科医师学会（American College of Chest Physicians, ACCP）^[49]^认为LDCT筛查是个复杂而相互影响的过程，目前的重点应放在如何使筛查更广泛的应用上。美国癌症综合网络（National Comprehensive Cancer Network, NCCN）^[50]^从患者的角度对什么样的人应该筛查，筛查的风险，以及筛查流程的给出指导。各肺癌组织筛查指南的共同点都是为了将LDCT更有效的应用于肺癌防治。但截至到目前，美国预防服务工作组（USPSTF）尚未更新其肺癌筛查指南。

## 4 问题与展望

NLST得出的LDCT肺癌筛查降低20%肺癌死亡的结果结束了多年来能否降低肺癌死亡率的争议，这项研究在肺癌筛查和早期诊断领域具有里程碑式的意义。基于NLST的结果，多个医学组织已经修正了肺癌筛查的指南。但低剂量螺旋CT筛查仍有一些问题需要进一步研究，如哪些人群最能从筛查中获益？筛查频率降低是否会影响筛查的效果？高危人群及个体需要接受多少次筛查？此外，过高的假阳性和成本效益也是在制定肺癌筛查政策前需要考虑的问题。

我们相信，随着LDCT肺癌筛查方法的不断完善，分子生物学的发展及新的敏感且特异的肺癌筛查早诊标志物在肺癌早期诊断中的应用，以及低剂量螺旋CT与分子标志物在肺癌筛查早诊中的联合应用，必将为肺癌的筛查和早诊带来新的希望。
